# Movie Events Detecting Reveals Inter-Subject Synchrony Difference of Functional Brain Activity in Autism Spectrum Disorder

**DOI:** 10.3389/fncom.2022.877204

**Published:** 2022-05-03

**Authors:** Wenfei Ou, Wenxiu Zeng, Wenjian Gao, Juan He, Yufei Meng, Xiaowen Fang, Jingxin Nie

**Affiliations:** ^1^Guangdong Key Laboratory of Mental Health and Cognitive Science, Center for Studies of Psychological Application, School of Psychology, South China Normal University, Guangzhou, China; ^2^Key Laboratory of Brain, Cognition and Education Sciences, Ministry of Education, South China Normal University, Guangzhou, China; ^3^Dongcheng Central Primary School, Dongguan, China

**Keywords:** movie-watching fMRI, sliding windows, inter-Subject correlation (ISC), inter-Subject function correlation (ISFC), autism spectrum disorder (ASD)

## Abstract

Recently, movie-watching fMRI has been recognized as a novel method to explore brain working patterns. Previous researchers correlated natural stimuli with brain responses to explore brain functional specialization by “reverse correlation” methods, which were based on within-group analysis. However, what external stimuli drove significantly different brain responses in two groups of different subjects were still unknown. To address this, sliding time windows technique combined with inter-Subject functional correlation (ISFC) was proposed to detect movie events with significant group differences between autism spectrum disorder (ASD) and typical development (TD) subjects. Then, using inter-Subject correlation (ISC) and ISFC analysis, we found that in three movie events involving character emotions, the ASD group showed significantly lower ISC in the middle temporal gyrus, temporal pole, cerebellum, caudate, precuneus, and showed decreased functional connectivity between large scale networks than that in TD. Under the movie event focusing on objects and scenes shot, the dorsal and ventral attentional networks of ASD had a strong synchronous response. Meanwhile, ASD also displayed increased functional connectivity between the frontoparietal network (FPN) and dorsal attention network (DAN), FPN, and sensorimotor network (SMN) than TD. ASD has its own unique synchronous response rather than being “unresponsive” in natural movie-watching. Our findings provide a new method and valuable insight for exploring the inconsistency of the brain “tick collectively” to same natural stimuli. This analytic approach has the potential to explore pathological mechanisms and promote training methods of ASD.

## Introduction

Naturalistic stimuli, such as movies, audio stories, and virtual reality are increasingly used as stimuli in neuroscience research (Zhang et al., 2021). Naturalistic paradigms are more ecological fMRI designs that elicit neural processes of the whole brain in rich spatiotemporal contexts (Hasson et al., 2004; Sonkusare et al., 2019). Movie-watching activates a large part of the human brain, triggering a common response across individuals (Khosla et al., 2021). Since usually a very small portion of the brain’s overall activity can be identified in traditional task designs paradigm (Kauppi et al., 2017), it may be challenging to predict how findings generalize to more complex ecological stimulus conditions (Salmi et al., 2013). Meanwhile, owing to the simplicity of resting-state fMRI, it is difficult to establish relationships between BOLD signals and ongoing mental processes (Gonzalez-Castillo et al., 2021). Resting-state fMRI and task-based fMRI do not mimic real-world experiences, where the brain constantly receives massive amounts of rich sensory stimuli (Kauppi et al., 2017). Movie-watching fMRI scanning requires participants to integrate perceptual and cognitive systems to follow the complexity of movie plots and thus resemble situations of daily life (Lyons et al., 2020), which is difficult to attain in resting-state fMRI and task-based fMRI. Moreover, task-based fMRI cannot avoid the problem of poor data quality caused by head motion, especially when collecting data from children with neurodevelopmental disorders (Pua et al., 2020). Some researchers found significantly lower head motion when watching a movie compared with performing a task and resting e.g., (Cantlon and Li, 2013; Vanderwal et al., 2015). Movie-watching fMRI scanning lowers head motion of participants, easing children’s studies (Bolton et al., 2020).

To model brain activities during movie-watching, Hasson et al. (2004) introduced inter-Subject correlation (ISC) analysis method by calculating the Pearson correlation coefficient of time series in corresponding brain regions between two participants, describing the similarity of inter-Subject functional activity in same brain regions induced by external stimuli. Simony et al. (2016) extended ISC by introducing inter-Subject functional correlation (ISFC), which provides inter-regional synchronous information by calculating the correlations between time series of one brain region in one participant and time series of all brain regions in other participants. However, most analyses using ISC and ISFC often remain at a static level, where only one estimate is obtained from full duration of movie-watching (Finn et al., 2018; Tei et al., 2019; Salmi et al., 2020). As movies involve an ever-changing sequence of time-overlapping cues that the brain adjusts dynamically, one estimate during the whole movie may not accurately describe how the brain changes over time and probably is impossible to pinpoint which movie cues drive brain fluctuation (Bolton et al., 2020). Recently, the dynamic inter-Subject analysis is an emerging frontier in naturalistic paradigms (Bolton et al., 2018, 2020; Yang et al., 2020). Dynamic ISFC tracks whole-brain functional connectivity changes over time in each sliding time window, so we can establish a relationship between movie cues and time-varying brain connectivity (Bolton et al., 2019).

To identify relationships between movie events and specific brain regions, reverse correlation analysis is widely adopted in movie-watching fMRI analysis (Hasson et al., 2004; Jääskeläinen et al., 2008; Wagner et al., 2016; Richardson et al., 2018). For instance, Hasson revealed that the fusiform sulcus is activated mainly by close-ups of face images, whereas the collateral sulcus is mostly activated by images of indoor and outdoor scenes (Hasson et al., 2004). Wagner revealed that the dorsal medial prefrontal cortex is driven primarily by social interaction scenes (Wagner et al., 2016). Hilary Richardson found that the theory of mind network synchrony is induced by movie scenes depicting characters’ beliefs, desires or emotions, and the pain network is correlated with movie events showing characters’ physical pain (Richardson et al., 2018). In these reverse correlation analyses, certain brain regions are selected as ROI based on prior researches, and each ROI’s average time series is z-normalized. Then the signal values across subjects for each time point are tested using a one-tailed *t*-test. Events are defined as two or more consecutive significantly positive time points. These methods are conducted by within-group of subjects and may be difficult to detect movie events with potential group differences in neural activity, which is important in clinical applications.

Thus, in order to identify movie events with different neural activity patterns in different groups, we proposed a novel approach combining sliding time windows technique with ISFC. We hypothesized that movie events with significant group differences could be identified by calculating the number of significant ISFC difference between groups in a range of sliding time windows. Then we compared ISC and ISFC responses during these identified movie events to explore if they have different brain activity patterns.

The proposed method was applied to find brain activity patterns of autism spectrum disorder (ASD) in this study. ASD is a neurodevelopmental disorder characterized by impairments in social interaction and communication as well as restricted repetitive behaviors and interests (Whitehouse et al., 2021). Studying brain activity of ASD versus control subjects in naturalistic stimuli with social interaction portrayed in movies may enhance understanding of how the brain is functioning in real life (Salmi et al., 2013). From a clinical point of view, naturalistic paradigms provide novel insights into investigations of brain dysfunction through detecting changes in neuropsychiatric disorders (Wang et al., 2017).

## Materials and Methods

### Subjects

The imaging data were obtained from Child Mind Institute Healthy Brain Network.^1^ Our selection criteria were as follows: (1) 6–17 years of age; (2) scanned T1-weighted images, resting-state images and movie-watching fMRI; (3) image coverage over 90% of the brain; (4) no history of other psychiatric or emotional disorders. These criteria left us with a well-matched dataset of 34 typically developmental children and 34 participants with ASD. There were no significant group differences in age and gender as shown in Table 1.

**TABLE 1:** Demographics of the participants.

	ASD (*n* = 34)	TD (*n* = 34)	*p*
Gender(male/female)	28/6	22/12	0.09^a^
Age	11.28 ± 3.30	11.12 ± 2.73	0.83^b^
WISC	99.56 ± 18.91	103.06 ± 14.87	0.40^b^
SCQ	13.18 ± 5.54	5.62 ± 3.29	< 0.05^b^
ASSQ	19.18 ± 8.40	2.41 ± 2.94	< 0.05^b^

*^a^Indicates p-value for χ^2^ test.*

*^b^Indicates p-value for two-sample t-test.*

*There are no significant group differences in age, gender, and WISC. There are significant group differences in SCQ and ASSQ.*

*WISC, Wechsler Intelligence Scale; SCQ, social communication questionnaire; ASSQ, autism spectrum screening questionnaire.*

### Image Acquisition

All images were acquired from 3T magnetic resonance imaging (MRI) scanners using a Siemens 32-channel head coil, collected at Rutgers University Brain Imaging Center and CitiGroup Cornell Brain Imaging Center, both using the same MRI scan parameters. T1 image scanning parameters were as follows: 224 axial slices; 0.8 mm^3^× 0.8 mm^3^× 0.8 mm^3^ resolution; TR = 2,500 ms; TE = 3.15 ms; flip angle = 8. Functional images were obtained while the subjects watched a 10-min clip of *Despicable Me* (from 1:02:09 to 1:12:09) with an echo-planar imaging (EPI) sequence (60 axial slices; 2.4 mm^3^× 2.4 mm^3^× 2.4 mm^3^ resolution; TR = 800 ms; TE = 30 ms; flip angle = 31).

The movie roughly describes that a bad guy Gru adopts three little girls to achieve his plan of stealing the moon. He develops an emotional connection in the time of telling stories and playing together with them until Dr. Nefalio sends the girls away to prevent them from influencing their plan of stealing the moon, which makes Gru feel lost.

### Data Preprocessing

Data preprocessing was performed via Data Processing Assistant for Resting-state fMRI: DPARSF v5.0^2^ toolbox (Yan et al., 2016). The first 10 time points from functional images were discarded to ensure a steady-state longitudinal magnetization, and then the remaining consecutive volumes were performed alignment correction. Structural and functional MRI images were co-registered after skull-stripped. T1 images were segmented into gray matter (GM), white matter (WM) and cerebrospinal fluid (CSF), and normalized to Montreal Neurological Institute (MNI) space. These transformation parameters were then applied to functional images. The normalized images were resampled to 3 × 3 × 3 mm^3^. The fMRI signals were further linear detrended and retained between frequency band of 0.01 and 0.1 Hz by a bandpass filter. Finally, Friston 24 head motion parameters as well as GM, WM, and CSF signals were regressed out as nuisance covariates. Furthermore, the whole brain was divided into 200 ROIs based on 200-ROI parcellations template (Craddock et al., 2012) and then average time series of 200 ROIs of all subjects were extracted.

### Inter-Subject Correlation and Inter-Subject Function Correlation

**FIGURE 1:**
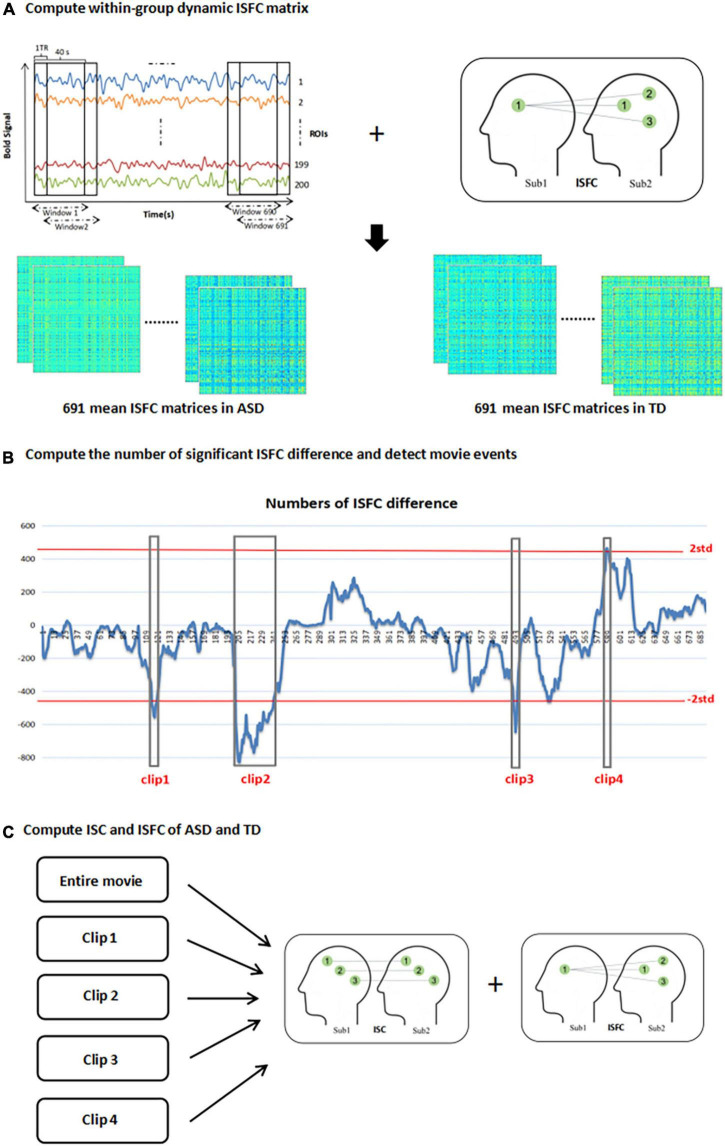
Depiction of methods. **(A)** segment the time series of 200 ROI into a series of overlapping windows with length 40s and step of 1 TR. calculate mean ISFC matrix of 691 time windows in ASD and TD group; **(B)** compute the number of significant ISFC difference between two groups in each time window and then detect movie events [the horizontal axis represents the windows and the vertical axis represents the numbers of ISFC difference between two groups (positive value means that significant ISFC of ASD > TD is greater than significant ISFC of ASD < TD, negative value is opposite), and the red lines are the two standard deviations thresholds for detecting movie clips with significant differences]; **(C)** calculate the ISC and ISFC of ASD and TD in entire movie and four movie events.

ISFC calculates the Pearson correlation coefficient between response time courses in one brain region of one subject and all other brain regions of other subjects (Simony et al., 2016). Doing this analysis across subjects ensures that the correlations only originate from movie stimuli, but not from non-neuronal contributions including head motion, physiological noise, or scanner artifacts (De Blasi et al., 2020; Jimenez et al., 2020). Since the full ISFC matrix was not symmetric, we transformed it to a symmetrical matrix given by $\frac{1}{2}\,\left(\text{ISFC}+{\text{ISFC}}^{T}\right)$ according to Simony’s research (Simony et al., 2016). The diagonal of this group-based ISFC matrix is ISC, which is the correlation between the same ROIs across subjects. We calculated the global ISFC and ISC of ASD and typical development (TD), respectively, when watching the whole movie and different movie clips (see Figure 1C), to illustrate the effect of different stimuli on the whole brain correlation between two groups.

### Movie Events Detection

Average time series of 200 ROIs in all subjects were divided into several overlapping windows with a length of 40s and step of 1 TR using sliding windows technique. The whole movie was ultimately divided into 691 windows. Each time window contained *n*×(*n*−1) pairwise ISFC matrices in one group (*n* means subject numbers in one group, *n* = 34 in our study). Then the mean ISFC matrix of each time window of two groups of subjects was calculated, respectively, which represented within-group mean functional connectivity strength of the group in each time window (see Figure 1A). Permutations test (10,000 times) was performed on each connection and family-wise error (FWE) correction was used to find significantly different ISFC between ASD and TD in each time window, attaining the result of significant ISFC of ASD > TD and significant ISFC of ASD < TD, respectively. Then the number of significant ISFC of ASD > TD minus significant ISFC of ASD < TD was calculated for each time window (for example, in the first time window, the number of significant ISFC of ASD > TD is 0, the number of significant ISFC of ASD < TD is 12, so the number of significant ISFC of ASD > TD minus significant ISFC of ASD < TD is -12). We attained 691 numbers represented inter-group significant ISFC differences in 691 time windows. The number series for 691 time windows was transformed into a normal distribution with standard deviation of one and time windows with the number larger or smaller than two standard deviations (*p* < 0.0456) were retained, which were identified as the inter-group different movie events (see Figure 1B).

### Statistics Analysis

Group differences of each ISFC and ISC were tested by subject level permutations test (10,000 times) method. The null hypothesis is that ASD and TD have the same effect of different stimuli on synchrony of the brain functional activity. After permutation test, we got the difference distribution of two groups, and then we calculated *p*-value of the real difference value of ISFC/ISC in the whole distribution. The significant ISC was considered after corrected for multiple comparisons with False Discovery Rate (FDR) correction and *p*-value was still less than 0.05. The significant ISFC map was derived after corrected for multiple comparisons with FWE correction and *p*-value was still less than 0.05. The above steps were performed in the condition of watching the whole movie and watching detected movie clips.

## Results

### Detected Movie Clips

According to the above method, four movie clips were detected (three or more consecutive time windows) with significant group differences for subsequent analysis (as shown in Figure 1B). Clip 1 was from window 115 to window 120 (time: 1:39–2:23); clip 2 was from window 202 to window 242 (time: 2:49–4:01); clip 3 was from window 491 to window 494 (time: 6:40–7:22) and clip 4 was from window 587 to window 589 (time: 7:57–8:38). In the first three clips, the brain responses of TD were greater than that of ASD, but clip 4 was the opposite.

Clip 1 focused on a warm and relaxing scene that Gru told a bedtime story to three little girls. Clip 2 depicted a complex sequence of scenes: the girls showed their frustration when Gru rejected their request for a goodnight kiss; Gru smiled when seeing the girls’ portrait; Gru exaggerated to hide his true desire to see the girls’ dance performance. Clip 3 told that after three little girls were sent away, the minions shed tears, Gru was emotionally lost, and the little girls were also very sad. Clip 4 showed a series of preparations for Gru before landing on the moon: riding a lift plate, entering a capsule, operating instruments, etc.

### Inter-Subject Correlation Under Natural Stimulation

**FIGURE 2:**
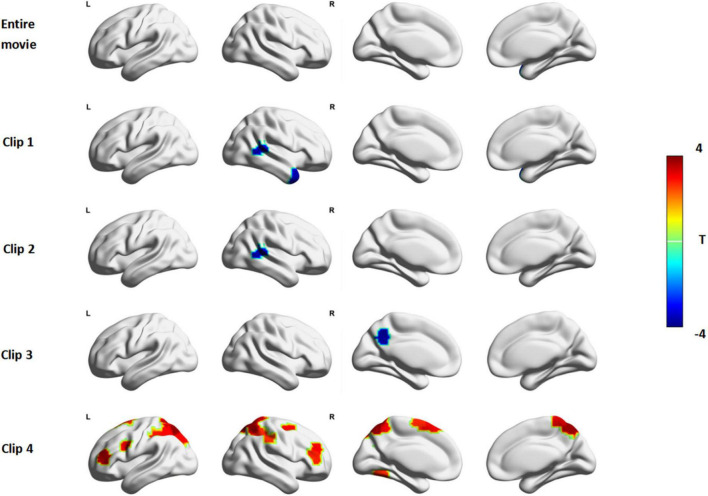
The ISC difference between ASD and TD in *Despicable Me*. The red color indicates that the ISC in ASD is significantly larger than that in TD and the blue color indicates that the ISC in ASD is significantly lower than that in TD (permutations test, FDR, *p* < 0.05). Entire Movie represents watching the whole ten-minute movie; Clip 1, 2, 3, and 4 denotes watching the movie clip 1, 2, 3, and 4, respectively.

ISC was used to characterize patterns of brain activation during shared response time courses between two groups. Inter-group differences of ISC were conducted to examine whether the patterns of synchronous activation differed in different movie events. We found no significant differences between ASD and TD in brain synchrony during the full clip of *Despicable Me* (Figure 2, row1). The synchrony of some brain regions in ASD was less than TD in clip 1, clip 2 and clip 3. But in clip 4 many brain areas of significantly greater synchrony were observed in the ASD group.

In clip1, the ASD group showed significantly lower ISC than the TD group (Figure 2, row2) in the right middle temporal gyrus (MTG.R), right temporal pole (TPO.R) and cerebellum. In clip 2, ISC of the right middle temporal gyrus (MTG.R) and left caudate (CAU.L) of ASD showed less synchronous activity than that of TD (Figure 2, row3). In clip3, compared with TD, the decreased ISC of the left precuneus (PCUN.L) and cerebellum was observed in ADHD (Figure 2, row4).

Based on Yeo-7 Networks parcellation (Yeo et al., 2011), we divided 200 ROIs into seven brain cortical networks as follows: visual network (VN), frontoparietal network (FPN), default mode network (DMN), sensorimotor network (SMN), dorsal attention network (DAN), ventral attention network (VAN) and limbic network (LN; as shown in Supplementary Materials). During clip 4, ISC in ASD was stronger than that in TD focusing on the DAN (Figure 2, row5): right superior parietal gyrus (SPG.R), left superior parietal gyrus (SPG.L), right inferior parietal gyrus (IPG.R), left inferior parietal gyrus (IPG.L), right precuneus (PCUN.R), left precuneus (PCUN.L), right superior frontal gyrus (SFG.R), left middle occipital gyrus (MOG.L). And some regions in the ventral attention network including the right middle frontal gyrus (MFG.R), right supraMarginal (SMG.R), left supplementary motor area (SMA.L) had greater synchrony in ASD. In addition, the ISC of left precentral (PreCG.L), right postcentral (PoCG.R), left triangle inferior frontal gyrus (IFGtriang.L), right middle frontal gyrus (MFG.R), left fusiform (FFG.L) in ASD was also obviously greater than that in TD.

### Inter-Subject Functional Correlation Under Natural Stimulation

In terms of the differences of ISFC, a large majority of connections showed more responsive in TD than that in ASD from the results of the entire movie and all clips except clip 4. In clip 4, the ASD group showed more significantly increased ISFC than the TD group.

**FIGURE 3:**
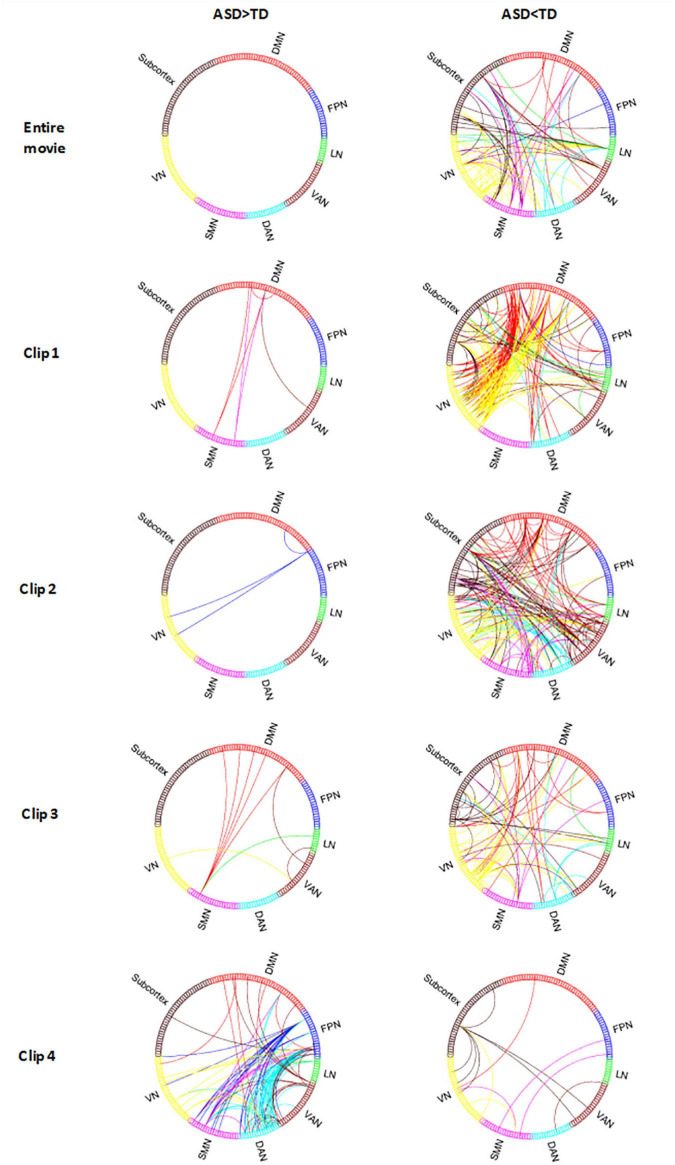
The ISFC difference between ASD and TD in *Despicable Me*. ASD > TD indicates the ISFC of ASD is significantly greater than that of TD. ASD < TD indicates the ISFC of ASD is significantly less than that of TD (permutations test, FWE, *p* < 0.05). Entire Movie represents watching the whole ten-minute movie; Clip 1, 2, 3, and 4 denotes watching the movie clip 1, 2, 3, and 4, respectively. VN, visual network; FPN, frontoparietal network; DMN, default mode network; SMN, sensorimotor network; DAN, dorsal attention network; VAN, ventral attention network; LN, limbic network; Subcortex, subcortex of brain.

In the entire movie, the brain regions of TD associated with the most responsive connections were located in the visual network (Figure 3, row1): right lingual (LING.R), left lingual (LING.L), right middle occipital gyrus (MOG.R), left middle occipital gyrus (MOG.L), right inferior occipital gyrus (IOG.R), left inferior occipital gyrus (IOG.L); the SMN: right postcentral gyrus (PoCG.R), left postcentral gyrus (PoCG.L), right superior frontal gyrus (SFG.R); the limbic network: left orbital inferior frontal gyrus (ORBinf.L).

In clip 1, ISFC in TD between the visual network: left calcarine (CAL.L), right fusiform gyrus (FFG.R), MOG.R, MOG.L, IOG.R, IOG.L and default mode network: left precuneus (PCUN.L), left medial superior frontal gyrus (SFGmed.L), left middle frontal gyrus (MFG.L) was obviously greater than that in ASD. The Limbic network: TPOmid.R, TPOmid.L also had stronger responsive connections with other brain areas (as shown in Figure 3, row2).

In clip 2, TD showed significantly increased ISFC in the whole brain network except the FPN (as shown in Figure 3, row3). Different from the entire movie and clip 1, the ventral attention network showed more connections in clip 2, with the majority of them involving right supraMarginal (SMG.R), left supraMarginal (SMG.L), right middle temporal gyrus (MTG.R) and right insula (INS.R). Additionally, the left fusiform gyrus (FFG.L) of the limbic network and cerebellum also showed increased ISFC with other networks.

In clip 3, TD also displayed increased ISFC pattern in the whole brain network as in condition of the entire movie, with the majority of regions involving IOG.R, IOG.L, SMA.L, PCUN.L and cerebellum (Figure 3, row4). The Limbic network (ORBinf.L, TPOmid.R, TPOmid.L) of ASD, which was significantly different from TD in the entire movie and clip 1, was also found in clip 3.

In clip 4, ISFC in ASD was more strongly detected between the FPN (MFG.R, IPG.R, SFGmed.L, IFGtriang.L) and DAN (SPG.R, SPG.L, IPG.LR, IPG.L, PCUN.R, PCUN.L), the FPN and SMN (PoCG.R, PCL.L) than that in TD (as shown in Figure 3, row5).

## Conclusion and Discussion

Using movie event detecting analysis, our study has different findings compared with previous studies. ASD was not “unresponsive” to diverse stimuli all the time compared to TD subjects. The ISC in the DAN and VAN, as well as ISFC between the FPN and DAN, FPN and SMN in ASD was significantly increased under specific movie contents. Movie events detecting in movie-watching fMRI has the potential to explore pathological mechanisms and promote training methods for psychiatric disorders.

### Brain Activity Desynchronized Between Autism Spectrum Disorder Subjects During Naturalistic Stimuli

ISC analysis revealed that ASD displayed different neural responses in social scenes during movie-watching compared with TD. ISC has the potential to track attention differences between different individuals. The decreased ISC suggests reduced functional specialization of corresponding brain regions to natural stimuli (Moraczewski et al., 2018). Consistent with previous studies, we found that the brain regions desynchronized in ASD during watching movie contents with various types of social interactions (Hasson et al., 2009; Salmi et al., 2013; Byrge et al., 2015; Lyons et al., 2020). The ASD group showed significantly lower ISC than the TD group in several brain areas, including the right middle temporal gyrus (MTG.R), right temporal pole (TPO.R), left caudate (CAU.L), left precuneus (PCUN.L) and cerebellum. It meant that neural responses of the brain areas in ASD were more variable or individualized while watching clip1, clip2, clip3. Our results were consistent with previous findings that the middle temporal gyrus is critical in processing social signals (Carrington and Bailey, 2009) and the temporal pole is highly correlated with memory of episodic events and emotional regulation (Gosnell et al., 2020). Precuneus is considered as part of the theory of mind network or mentalizing network and is activated when people infer mental states of others (Takahashi et al., 2015; Yeshurun et al., 2017). Caudate is one of the brain regions in the emotional-motivational system (Loonen and Ivanova, 2016). Thus, our findings supported that ASD shows desynchronized brain activity when processing social information.

In addition to ISC analysis, we also measured the impact of movie stimuli on inter-Subject functional connectivity. We found that ASD maintained decreased brain network connections in the entire movie and movie clip1, clip2 and clip3, especially in the visual network, default mode network, ventral attention network and limbic network. Social visual engagement difficulties in ASD, performed as a reduced preference for social stimuli, which were reported in many studies (Falck-Ytter et al., 2018; Lombardo et al., 2019). Lombardo et al. indicated ASD shows functional hypoconnectivity between the default mode network and visual or attention networks compared to TD, and similar results were observed in clip1, clip2 in our study. The default mode network associated with social inferences and understanding of others has been found predominantly connectivity reduction in ASD (Hong et al., 2019; Chen et al., 2021). In addition, the limbic network plays a role in emotion and memory function. The temporal pole, as a part of the limbic network and processing face recognition, autobiographical memory, and semantic memory, was atypical in ASD (Chen et al., 2017; Li et al., 2020). By detecting movie events, we found reduced functional connections of the visual network, default mode network, and limbic network in ASD during watching movie clips with a social interaction plot. Our research confirmed previous results which showed decreased inter-network functional activation of the above networks in resting-state fMRI and task-based fMRI.

### High Brain Activity Synchronized Between Autism Spectrum Disorder Subjects During Special Stimuli

Specifically, our results showed that ASD displayed extraordinarily increased brain synchronous activity under the detected movie clip 4, which were inconsistent with previous researches. A possible explanation was that predecessors’ results were based on static analysis of the entire movie, but we took more consideration into specific characteristics of the detected movie clips. The movie clip 4 showed a series of preparations for Gru before landing on the moon, focusing on objects and scenes shot. We found that ASD was associated with increased ISC across the whole brain in watching a movie clip that depicted none social cognition cues. The highly synchronized brain areas were mainly observed in the DAN and ventral attention network. The DAN plays a role in externally oriented attention, such as top-down attentional control (Corbetta and Shulman, 2002). The ventral attention network modulates bottom-up attentional control (Corbetta et al., 2008). High consistency in two attention networks observed in ASD might suggest that ASD participants were more interested in and consistently attended to movie contents presented in clip 4. Our study suggested that ASD had a unique synchronous response pattern under the case of natural stimulation focusing on objects shot, rather than being “unresponsive” all the time.

Other remarkable discoveries in our results suggested that ISFC between the FPN and DAN, FPN and SMN in ASD were increased during watching movie clip 4. The FPN is involved in cognitive control (Markett et al., 2014), and the DAN is associated with attentional control, modulating goal-oriented attention (Corbetta and Shulman, 2002). ASD-specific increases in ISFC for a connection linking the FPN and DAN reflected the arousal and attentional impact of the movie scenes. This result might reveal that the brain activity of ASD is not “unresponsive” to diverse stimuli, but requires specific materials to attract their attention.

### Possible Clinical Application of Detecting Movie Events

Naturalistic stimuli induce reproducibly spatiotemporal brain responses, elicit the essence of many perceptual and cognitive processes (Zhang et al., 2021), and can capture the complex and nuanced character of social cognition (Hari et al., 2015). The clinical application is promising for mapping and probing functional brain patterns in clinical patients (Eickhoff et al., 2020). A series of researches have revealed abnormal brain functional connectivity or functional synchrony in clinical patients during movie watching. Patients with melancholia show less functional connectivity in the ventral medial prefrontal cortex, anterior cingulate cortex (ACC) and superior temporal gyrus in natural-viewing conditions (Guo et al., 2016). Salmi et al. found that several areas in attention networks and sensory cortices are significantly desynchronized in attention-deficit/hyperactivity disorder when watching a movie clip (Salmi et al., 2020). K.M. Lyons revealed ASD have significantly less neural synchrony in brain regions related to “theory of mind” while watching the movie *Despicable Me* (Lyons et al., 2020). These advanced discoveries have accelerated maturation of movie-watching fMRI for pathological research. Using dynamic sliding windows technique and ISFC method, we caught the number of significant ISFC between groups to identify the significantly powerful brain activation between different groups during watching the same movie. We found no brain synchronous differences in ISC during watching a ten-minute movie of *Despicable Me*. Maybe the movie spans a long time, so the brain’s state transitions cannot be captured. Therefore, compared with the entire movie, detected movie events can better reveal the activity pattern of the brain corresponding to time-varying stimuli to better explore the pathological mechanisms of psychiatric disorders.

In addition to looking for pathology, movie events detecting approach has its potential to promote training methods of clinical patients such as ASD. Previous studies have introduced many helpful training methods for ASD. Gaze-contingent training effectively increases attention toward the face of onscreen social characters in ASD (Wang et al., 2020). Phung and Goldberg (2019) found that martial arts training improves executive functioning deficits in children with ASD. Yu et al. indicated that game-based exercise training program promotes physical and mental health in children with ASD (Yu et al., 2018). Besides, a study identified that peer training intervention increases initiations and responses in students with ASD (Owen-DeSchryver et al., 2008). However, previous studies were based on behavioral aspects, and there were few studies to explore ASD training methods in cognitive neuroscience. Our movie-watching fMRI results found that increased ISFC in the FPN and DAN of ASD in clip 4, might suggest movie contents attract ASD’s attention and promote executive control function of ASD. When audiences watch a movie, the genre of movie and the certain scenes of clips attract their attention. Using movie events detecting, we can better identify what movie contents are different clinical patients interested in. Through exploring which stimulus materials are more likely to stimulate stronger brain activity, it is possible to exercise cognitive control with these stimulus materials in ASD.

## Limitations

This study has some limitations. First, although abnormal brain areas we found in ASD is consistent with previous studies, only one movie was analysed in our study. It may introduce potential bias for limited movie style or contents. Second, ROI-level analysis was conducted in our research, which increased signal-to-noise ratio (SNR) and decreased computational cost (Korhonen et al., 2017). But it also might lose some valuable information by averaging the BOLD signals of its voxels. In future research, it is possible to explore inter-Subject synchrony difference of functional brain activity at more fine-grained level (such as voxel-level). Third, if the research could be repeated using different datasets, the results would be more robust.

## Data Availability Statement

The datasets presented in this study can be found in the Child Mind Institute Healthy Brain Network (http://fcon_1000.projects.nitrc.org/indi/cmi_healthy_brain_network/).

## Ethics Statement

Ethical review and approval was not required for the study on human participants in accordance with the local legislation and institutional requirements. Written informed consent to participate in this study was provided by the participants’ legal guardian/next of kin.

## Author Contributions

JN conceived the project. WO processed and analyzed fMRI data. WO and WZ wrote the manuscript. WG wrote the code. JH, YM, and XF revised the manuscript. All authors contributed to the article and approved the submitted version.

## Conflict of Interest

The authors declare that the research was conducted in the absence of any commercial or financial relationships that could be construed as a potential conflict of interest.

## Publisher’s Note

All claims expressed in this article are solely those of the authors and do not necessarily represent those of their affiliated organizations, or those of the publisher, the editors and the reviewers. Any product that may be evaluated in this article, or claim that may be made by its manufacturer, is not guaranteed or endorsed by the publisher.

## Footnotes

1
http://fcon_1000.projects.nitrc.org/indi/cmi_healthy_brain_network/

2
http://rfmri.org/DPARSF
