# Supplementary material for: Movie Events Detecting Reveals Inter-Subject Synchrony Difference of Functional Brain Activity in Autism Spectrum Disorder
Source: Front Comput Neurosci. 2022 May 3;16:877204. doi: 10.3389/fncom.2022.877204 (PMC9110681; doi:10.3389/fncom.2022.877204)
Supplement: Supplementary file 1 [file Data_Sheet_1.PDF]

Supplementary Material

1 The ROI to subnetwork mapping results

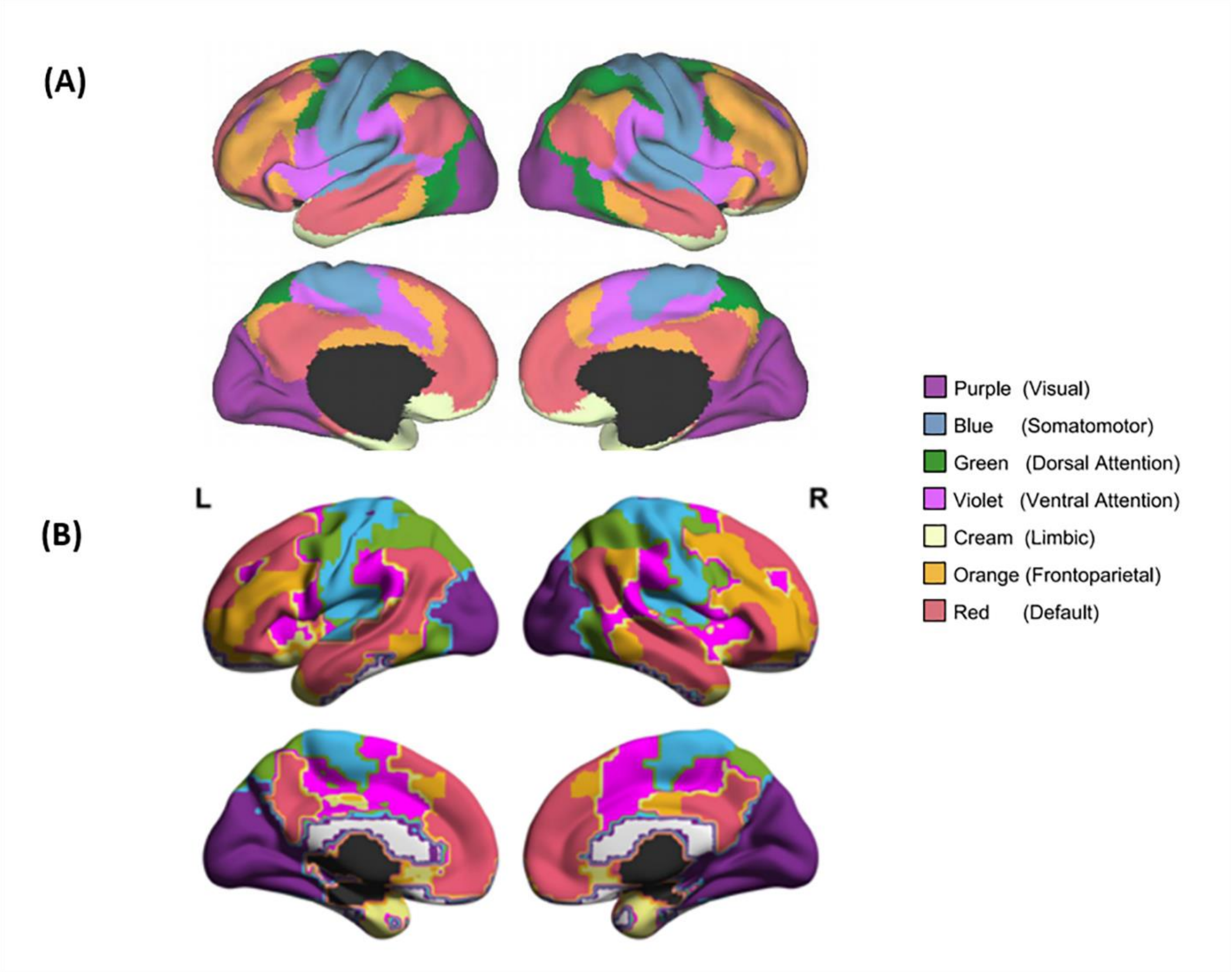

Supplementary Figure 1. (A) is Yeo-7 Networks mask. (B) is mapping results of 200 ROI divided to seven brain cortical networks in current study, which are as follows: visual network (VN), sensorimotor network (SMN), dorsal attention network (DAN), ventral attention network (VAN), limbic network (LN), frontoparietal network (FPN), default mode network (DMN).

2 The ROI to subnetwork labeling results

Supplementary Table 1. This table shows the ROI to subnetwork labeling results. Network indicates seven networks and subcortex. ROI indicates ROI number of 200-ROI parcellations. Label indicates the label of brain region corresponding to ROI number.

Network	ROI	Label	Network	ROI	Label
Visual network	1	Occipital Mid L	Sensorimotor network	8	Postcentral L
	3	Precuneus R		21	Paracentral Lobule L
	19	Calcarine L		24	Temporal Sup L
	26	Occipital Inf R		28	Postcentral R
	44	Calcarine R		35	Insula R
	54	Cuneus L		60	Precentral R
	62	Fusiform R		65	Postcentral R
	70	Calcarine L		66	Temporal Sup R
	81	Cuneus R		88	Postcentral L
	85	Temporal Mid R		90	Precentral L
	89	Calcarine R		96	Postcentral L
	97	Occipital Mid L		98	Postcentral R
	102	Occipital Mid R		111	Paracentral Lobule L
	105	Lingual R		121	Rolandic Oper L
	108	Calcarine L		123	Supp Motor Area L
	122	Fusiform L		134	Frontal Sup R
	131	Occipital Mid L		146	Temporal Sup L
	138	Lingual R		154	Postcentral L
	142	Occipital Sup R		157	Postcentral R
	147	Precuneus L		185	Rolandic Oper R
	150	Occipital Mid L		200	Temporal Sup L
	158	Occipital Inf L	Ventral attention network	4	Temporal Sup L
	159	Occipital Inf L		6	Cingulum Mid R
	170	Occipital Mid R		13	Supp Motor Area L
	172	Fusiform R		20	Insula L
	175	Occipital Inf R		33	SupraMarginal L
	177	Lingual L		55	Cingulum Mid L
	179	Lingual L		59	Insula R
	189	Fusiform L		69	Temporal Mid R
	195	Lingual R		76	Cingulum Mid L
	16	Temporal Mid L		79	Cingulum Mid L
	17	Frontal Inf Oper R		83	Insula R
	31	Parietal Sup R		116	SupraMarginal L
	50	Precentral L		119	Rolandic Oper R

Dorsal attention network	63	Temporal Inf L		125	Frontal Mid L
	64	Frontal Sup R		128	SupraMarginal R
	73	Precentral L		137	Insula R
	93	Parietal Inf R		161	Supp Motor Area R
	100	Temporal Inf R		165	Frontal Inf Oper L
	114	Occipital Mid L		168	Frontal Mid R
	132	Parietal Sup R		180	SupraMarginal R
	136	Parietal Sup L		182	Supp Motor Area L
	143	Temporal Mid R		184	Insula L
	156	Parietal Inf L	Frontoparietal network	7	Parietal Inf R
	163	Precuneus R		12	Frontal Sup R
	171	Parietal Inf L		23	Frontal Inf Tri L
	188	Parietal Sup L		25	Frontal Mid R
	197	Precuneus L		29	Cingulum Mid R
Limbic network	27	Fusiform L		34	Precentral L
	32	Temporal Pole Mid R		38	Frontal Mid R
	43	Temporal Inf L		39	Temporal Mid R
	57	Frontal Inf Orb L		42	Frontal Mid Orb L
	78	Temporal Pole Mid L		75	Frontal Mid R
	87	Fusiform R		99	Temporal Mid L
	110	Temporal Pole Sup R		113	Frontal Mid Orb R
	112	Insula L		115	Precentral R
	160	Olfactory L		124	Frontal Mid Orb R
	198	Fusiform R		127	Frontal Mid R
	2	Angular L		149	Frontal Sup Medial L
	5	Cingulum Ant L		151	Frontal Inf Tri L
	11	Temporal Mid L		164	Frontal Inf Tri R
	14	Angular R		169	Frontal Mid Orb L
	22	Cingulum Ant R		183	Frontal Mid L
	40	Cingulum Ant R		9	Cerebelum 6 L
	46	Cingulum Mid R		10	Cerebelum Crus1 R
	49	Temporal Mid R		15	Caudate R
	51	Frontal Med Orb L		18	Thalamus R
	53	Frontal Inf Orb R		30	None
	56	Parietal Inf L		36	Cerebelum Crus1 L
	58	Precuneus L		37	Thalamus L

Default mode network	61	Frontal Mid L	Subcortex	41	Cerebelum 6 R
	71	Frontal Inf Orb R		45	Thalamus L
	72	Temporal Mid L		47	Caudate L
	74	Frontal Inf Orb L		48	Hippocampus R
	82	Angular L		52	Cerebelum 9 L
	91	Frontal Sup Medial L		67	Putamen L
	95	Frontal Mid L		68	None
	101	Temporal Inf L		77	Cerebelum Crus1 R
	104	Frontal Sup Medial L		80	Cerebelum Crus1 L
	106	Frontal Mid R		84	Caudate L
	107	Temporal Sup R		86	Cerebelum 9 R
	109	Frontal Med Orb L		92	Hippocampus L
	117	Temporal Mid L		94	Caudate R
	129	Temporal Sup L		103	Cerebelum 6 L
	133	Frontal Sup Medial L		118	Cerebelum 6 R
	139	Frontal Sup Medial R		120	Cerebelum Crus2 L
	140	Temporal Mid R		126	Cerebelum Crus1 R
	141	Frontal Inf Tri L		130	None
	144	Frontal Inf Tri R		135	Caudate R
	153	Temporal Sup R		145	Hippocampus L
	166	Angular R		148	None
	167	Frontal Mid L		152	Vermis 4 5
	173	Supp Motor Area L		155	Hippocampus R
	174	Precuneus L		162	Cerebelum 4 5 L
	181	Temporal Pole Sup R		176	Vermis 8
	186	Frontal Sup Medial R		178	Putamen R
	187	Frontal Sup R		190	Cerebelum 3 R
	191	Frontal Sup L		192	Cerebelum Crus2 L
	193	Frontal Sup R		194	None
	196	Temporal Pole Sup L		199	ParaHippocampal L
